# The Influence of Pro-Inflammatory Factors on Sclerostin and Dickkopf-1 Production in Human Dental Pulp Cells Under Hypoxic Conditions

**DOI:** 10.3389/fbioe.2019.00430

**Published:** 2019-12-17

**Authors:** Klara Janjić, Mohammad Samiei, Andreas Moritz, Hermann Agis

**Affiliations:** ^1^Department of Conservative Dentistry and Periodontology, University Clinic of Dentistry, Medical University of Vienna, Vienna, Austria; ^2^Austrian Cluster for Tissue Regeneration, Vienna, Austria; ^3^Department of Endodontics, Faculty of Dentistry, Tabriz University of Medical Sciences, Tabriz, Iran

**Keywords:** dental pulp, hypoxia, inflammation, *in vitro* techniques, regeneration

## Abstract

Sclerostin (Sost) and dickkopf (Dkk)-1 are inhibitors of the Wnt signaling pathway that plays a role in regenerative processes. Hypoxia-based strategies are used for regenerative approaches, but the influence of hypoxia on Sost and Dkk-1 production in a pro-inflammatory environment is unclear. The aim of this study was to assess if pro-inflammatory molecules have an influence on Sost and Dkk-1 production in dental pulp cells (DPC) under normoxia and hypoxia. Human DPC were treated with interleukin (IL)-1β, tumor necrosis factor (TNF)α or transforming growth factor (TGF)β, with L-mimosine (L-MIM) or hypoxia or a combination. Sost and Dkk-1 mRNA and protein levels were measured with qPCR and western blot, respectively. TNFα, TGFβ, L-MIM, or combined treatment did not modulate Sost and Dkk-1. IL-1β downregulated Sost at the mRNA level. Hypoxia alone and together with inflammatory markers downregulated Dkk-1 at the mRNA level. Sost and Dkk-1 protein production was below the detection limit. In conclusion, there is a differential effect of hypoxia and IL-1β on the mRNA production of Sost and Dkk-1. Pro-inflammatory molecules do not further modulate the effects of L-MIM or hypoxia on Sost and Dkk-1 production in DPC.

## Introduction

The Wnt signaling pathway regulates regenerative processes in various tissues, including oral tissues (Seo et al., 2012). Sclerostin (Sost) and dickkopf (Dkk)-1 are the main inhibitors of the Wnt signaling pathway. With that, they are important regulators of the signaling activity. In dentistry, Sost plays a role in tooth development where it is produced by odontoblasts (Naka and Yokose, 2011), decelerates reparative dentinogenesis and contributes to dental pulp volume (Collignon et al., 2017), influences bone and cementum phenotypes (Kuchler et al., 2014) and is associated with senescence in dental pulp cells (DPC) (Ou et al., 2018). For periodontitis treatment, a monoclonal antibody against Sost has already been evaluated in a pre-clinical study (Taut et al., 2013). Based on the current knowledge, Sost is also considered an interesting target for therapy in endodontics. Dkk-1 is a contributor to dentin formation and mineralization (Han et al., 2011), it might play a role in root resorption (Zhu et al., 2013) and it is possibly connected to the inflammatory response and bone resorption in periapical lesions (Zhang et al., 2014). Hence, also Dkk-1 could be of interest as target in regenerative endodontic approaches.

Hypoxia-based strategies aim to improve angiogenesis in regenerative approaches. One approach is based on the idea to pre-condition cells with hypoxic conditions or hypoxia mimetic agents “to train” them for the hypoxic region of a defect where they are supposed to support regeneration (Janjić et al., 2018b). Diverse effects of hypoxia on Sost and Dkk-1 were discussed (Genetos et al., 2010; Chen et al., 2013; Guo et al., 2014). In human DPC, the hypoxia mimetic agent L-mimosine (L-MIM) downregulated *SOST* and hypoxia downregulated *DKK-1* mRNA production, but this effect could not be reproduced at protein levels, where SOST and DKK-1 were only produced marginally or not at all (Janjić et al., 2018a). Interleukin (IL)-1 (Weng et al., 2012; Ruscitti et al., 2015), tumor necrosis factor (TNF)α (Korkosz et al., 2014; Sebastian and Loots, 2017) and transforming growth factor (TGF)β (Loots et al., 2012; Al Shareef et al., 2018), markers of inflammation, are able to increase levels of Sost and Dkk-1.

Thus, we hypothesized that basal levels of Sost and Dkk-1 can be elevated with inflammatory markers, such that basal levels of Sost and Dkk-1 as well as effects of treatment with hypoxia mimetic agents or hypoxia would be detectable. The aim of the study is to test if pro-inflammatory molecules alone or together with hypoxic conditions have an impact on Sost and Dkk-1 production in human DPC. This knowledge will help to evaluate if Sost and Dkk-1 should be considered as pharmacological targets under inflammatory or hypoxic conditions, e.g. after dental trauma, when regenerative endodontic therapy is indicated.

## Materials and Methods

### Cell Culture

**Figure 1:**
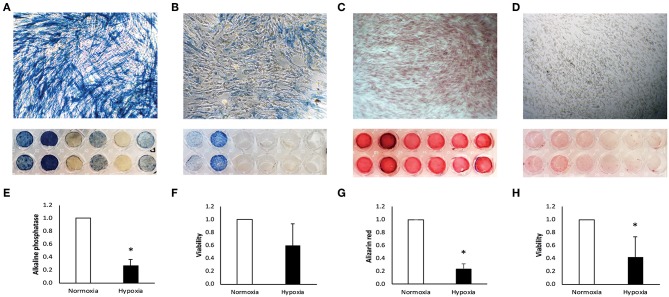
Characterization of human dental pulp cells (DPC). DPC under normoxia **(A)** and hypoxia **(B)** were stained for alkaline phosphatase. Staining was quantified and is displayed normalized to the normoxic control **(E)**. Metabolic activity under normoxia and hypoxia was measured in parallel after 7 days **(F)**. After 14 days, DPC were stained with alizarin red for calcium deposition under normoxia **(C)** and hypoxia **(D)** and quantified normalized to the normoxic control **(G)**. Metabolic activity was measured at the same time under normoxia and hypoxia **(H)**. Bars represent mean + standard deviation. *N* = 6. *p < 0.05 relative to normoxic control.

Human DPC were isolated from the dental pulp tissue of teeth that were extracted for orthodontic reasons and did not show any signs of inflammation. A detailed description of DPC isolation has already been published (Müller et al., 2015). Patients gave oral and written consent to their donation. Processing and use of patient material has been approved by the Ethical committee of the Medical University of Vienna, Vienna, Austria (#631/2007). DPC were cultured in α-minimal essential medium (αMEM medium; Gibco, Invitrogen Corporation, Carlsbad, CA, USA) including fetal bovine serum (FBS; Gibco) and antibiotics (penicillin, streptomycin and amphotericin; Gibco) at 37°C, 5% CO_2_ and 95% atmospheric moisture. DPC are adherent cells and appear spindle-shaped, corresponding to characteristic fibroblast morphology (Figure 1A). It has been reported that human DPC express surface markers for mesenchymal stem cells, thereby fulfilling the minimal criteria for mesenchymal stem cells (Rosowski et al., 2019).

For experiments, 50,000 DPC/cm^2^ in αMEM medium, supplemented with FBS and antibiotics were seeded into cell culture plates. DPC in passages 2–5 were used, harvested from 6 different donors (*N* = 6) at 80% confluency. Human DPC have an approximate doubling time of 1 day (Rosowski et al., 2019).

### Cell Treatment

DPC were treated 1 day after seeding for 24 h with the inflammatory markers IL-1β at 10 ng/ml (PeproTech Austria, Vienna, Vienna, Austria), TNFα at 10 ng/ml (PeproTech Austria) or TGFβ at 10 ng/ml (isoform TGFβ1; PeproTech Austria) in serum-free αMEM medium (Gibco) with penicillin and streptomycin (Gibco). The time frame of cell treatment was chosen to correspond to a biologically and clinically relevant situation where hypoxic conditions and inflammation occur due to e.g., trauma and normally should undergo regenerative therapy. Hypoxic conditions were mimicked with the hypoxia mimetic agent L-MIM at 1 mM or hypoxia at < 1% O_2_ was induced using the BD GasPak EZ Pouch system (Becton, Dickinson and Company, Franklin Lakes, NJ, USA) as it matches the biologically relevant range (Agata et al., 2008, 2013). The procedure was previously described in detail (Janjić et al., 2018a). DPC were additionally treated in combination with inflammatory markers and L-MIM or hypoxia, respectively. Untreated DPC under normoxia were used as control. All treatment solutions were prepared in serum-free αMEM medium with antibiotics. The choice of IL-1β (Weng et al., 2012), TNFα (Paula-Silva et al., 2009), TGFβ (Loots et al., 2012) and L-MIM (Janjić et al., 2018a) concentrations was based on previous publications.

### Alkaline Phosphatase Assay

DPC were cultured in αMEM medium containing 50 mmol/L L-ascorbic acid and 10 mmol/L β-glycerophosphate (Sigma-Aldrich) under normoxic and hypoxic conditions for 7 days. Hypoxia was induced as described above. Results on alkaline phosphatase production in DPC upon treatment with L-MIM were already published (Müller et al., 2015). A medium change was performed on day 5. For histochemical staining for alkaline phosphatase, DPC were fixed after 7 days with neutral buffered formaldehyde for 5 min at room temperature. A solution of Naphthol AS-TR phosphate disodium salt and Fast Blue BB Salt (Sigma-Aldrich) was added to fixed cells for 30 min at 37°C. Images of stained cells were taken under a light microscope at 100-fold magnification. A photometric measurement of the color change was performed at 405 nm.

### Alizarin Red Assay

DPC were cultured under same conditions as described for the alkaline phosphatase assay. Fixation with formaldehyde was performed after 14 days of culture, followed by staining with a 0.5 % solution of alizarin red (Sigma-Aldrich). Light microscopic images of the staining were taken at 100-fold magnification and color change was quantified at 450 nm with a photometer.

### Metabolic Activity Assay

A resazurin-based toxicity assay (Merck, Darmstadt, Germany) was performed as described by the manufacturer to evaluate metabolic activity of DPC cultured in αMEM medium containing 50 mmol/L L-ascorbic acid and 10 mmol/L β-glycerophosphate (Sigma-Aldrich) under normoxic and hypoxic conditions for 7 and 14 days. Fluorescence was measured in a photometer at an excitation wavelength of 540/34 nm and an emission wavelength of 600/40 nm.

### RNA Isolation and cDNA Synthesis

RNA was isolated from DPC 24 h after treatment with the RNeasy Plus Mini Kit (Qiagen, Hilden, NW, Germany) according to the protocol given by the manufacturer. The quality of isolated RNA was determined by the 260 nm/280 nm ratio and with a mean value of 2.036 ± 0.053 considered as pure material. RNA concentrations were measured photometrically and diluted to 1 μg for cDNA synthesis with a High Capacity cDNA Reverse Transcription Kit (Thermo Fisher Scientific, Waltham, MA, USA).

### qPCR

Produced cDNA was used to measure mRNA levels with TaqMan assays of *SOST* (Hs00228830_m1; Thermo Fisher Scientific, Waltham, MA, USA) and *DKK-1* (Hs00183740_m1; Thermo Fisher Scientific) by qPCR. Glyceraldehyde-3-phosphate dehydrogenase (*GAPDH*; Hs02758991_g1; Thermo Fisher Scientific) was used as reference gene. *GAPDH* showed a more stable mRNA expression in treated and untreated DPC than beta-actin and 18S rRNA, therefore it was chosen as reference gene. Data were analyzed with the 2^−ΔΔCT^ method.

### Western Blot

Total protein from DPC was extracted 24 h after treatment using Laemmli Sample Buffer (Bio-Rad Laboratories GmbH, Vienna, Austria). Protein on nitrocellulose membranes were incubated with primary antibodies against SOST (AF1406; R&D Systems, Inc., Minneapolis, MN, USA) and DKK-1 (MA5-32229; Thermo Fisher Scientific). GAPDH (MA5-15738; Thermo Fisher Scientific) was used as reference protein. Chemiluminescence was detected in a ChemiDoc MP System (Bio-Rad Laboratories GmbH).

### Statistics

Data are displayed as mean + standard deviation. The mean was calculated from results of six different donors (*N* = 6). The Kruskal-Wallis test and the Mann-Whitney test were used for statistical analysis and calculated with IBM SPSS Statistics Version 23 (IBM Corporation, Armonk, NY, USA). Statistical significance was determined as *p* < 0.05.

## Results

### Alkaline Phosphatase Assay

From six alkaline phosphatase DPC donors two donors did not respond under normoxia (Figure 1A) and only two stained positive for alkaline phosphatase under hypoxia (Figure 1B). Intensity of alkaline phosphatase staining was donor-dependent. Alkaline phosphatase production was significantly reduced under hypoxia, relative to the normoxic control (Figure 1E).

### Alizarin Red Assay

Positive staining for calcium deposition in alizarin red staining was found in all DPC donors under normoxia (Figure 1C), but only weak or no staining was found in DPC under hypoxia (Figure 1D). Calcium deposition was significantly reduced under hypoxic conditions, relative to normoxic conditions (Figure 1G).

### Metabolic Activity Assay

After incubation in αMEM medium containing 50 mmol/L L-ascorbic acid and 10 mmol/L β-glycerophosphate (Sigma-Aldrich) under normoxia and hypoxia, DPC under hypoxic conditions show a trend for reduced metabolic activity after 7 days (Figure 1F) and significant reduction after 14 days (Figure 1H).

### Sclerostin mRNA Levels

**Figure 2:**
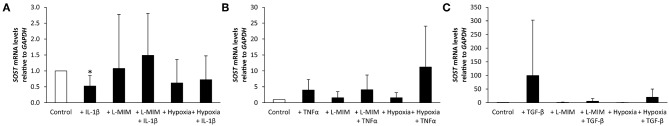
Sclerostin (*SOST*) mRNA levels in human dental pulp cells (DPC), normalized to the reference gene glyceraldehyde-3-phosphate dehydrogenase (*GAPDH*) and relative to the normoxic control (white bar). DPC were treated with different inflammatory markers [**(A)** interleukin-1beta (IL-1β), **(B)** tumor necrosis factor alpha (TNFα), **(C)** transforming growth factor beta (TGFβ)], a hypoxia mimetic agent [L-mimosine (L-MIM)], hypoxia, or their combinations (black bars). Bars represent mean + standard deviation. *N* = 6. *p < 0.05 relative to control.

*SOST* mRNA levels significantly decreased in DPC treated with IL-1β. L-MIM and hypoxia did not significantly modulate *SOST*. A combined treatment of IL-1β and L-MIM or hypoxia did not have any significant effect on *SOST* production. There was no significant difference in SOST production in DPC treated with L-MIM or hypoxia alone compared to combined treatment with IL-1β (Figure 2A).

*SOST* mRNA levels showed a trend to increase upon treatment with TNFα alone or combined with L-MIM or hypoxia, but this trend did not reach the level of significance. L-MIM and hypoxia did not show any significant modulation on *SOST* production. There was no significant difference in *SOST* production in DPC treated with L-MIM or hypoxia alone compared to combined treatment with TNFα (Figure 2B).

*SOST* mRNA levels showed a trend to increase upon treatment with TGFβ. L-MIM and hypoxia alone or in combination with TGFβ did not show any significant modulation on *SOST* production. There was no significant difference in *SOST* production in DPC treated with L-MIM or hypoxia alone compared to combined treatment with TGFβ (Figure 2C).

### Dickkopf-1 mRNA Levels

**Figure 3:**
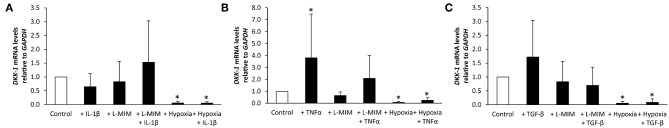
Dickkopf-1 (*DKK-1*) mRNA levels in human dental pulp cells (DPC), normalized to the reference gene glyceraldehyde-3-phosphate dehydrogenase (*GAPDH*), and relative to the normoxic control (white bar). DPC were treated with different inflammatory markers [**(A)** interleukin-1beta (IL-1β), **(B)** tumor necrosis factor alpha (TNFα), **(C)** transforming growth factor beta (TGFβ)], a hypoxia mimetic agent [L-mimosine (L-MIM)], hypoxia or their combinations (black bars). Bars represent mean + standard deviation. *N* = 6. *p < 0.05 relative to control.

*DKK-1* mRNA levels in DPC were not modulated when treated with IL-1β, L-MIM, or the combination of both. In contrast, DPC treated with hypoxia alone or hypoxia and IL-1β led to a significant downregulation of *DKK-1*. There was no significant difference in *DKK-1* production in DPC treated with L-MIM or hypoxia alone compared to combined treatment with IL-1β (Figure 3A).

*DKK-1* mRNA levels in DPC significantly increased upon treatment with TNFα. L-MIM and the combination of L-MIM and TNFα did not modulate *DKK-1*. Treatment with hypoxia alone or hypoxia and TNFα significantly decreased *DKK-1* production. There is no significant difference in *DKK-1* production in DPC treated with L-MIM or hypoxia alone compared to combined treatment with TNFα (Figure 3B).

*DKK-1* mRNA levels in DPC was not modulated when treated with TGFβ, L-MIM or the combination of both. DPC treated with hypoxia alone or hypoxia and TGFβ led to a significant downregulation of *DKK-1*. There was no significant difference in *DKK-1* production in DPC treated with L-MIM or hypoxia alone compared to combined treatment with TGFβ (Figure 3C).

### Sclerostin and Dickkopf-1 Protein Levels

No bands could be visualized for SOST or DKK-1 with western blot analysis in any of the DPC samples, including untreated controls (Supplementary Figure 1). Detection of GAPDH bands confirmed functionality of the method.

## Discussion

The current study aimed to raise production of Sost and Dkk-1 in human DPC with the inflammatory markers IL-1β, TNFα, and TGFβ, such that assessment of basal levels of Sost and Dkk-1 would be enabled as well as studying effects of hypoxic conditions on the production of Sost and Dkk-1.

A previous study revealed that basal levels of Sost and Dkk-1 proteins are hardly detectable in human DPC (Janjić et al., 2018a). Also in the current study, measurement of SOST and DKK-1 remains a major challenge. Current literature reports that pro-inflammatory markers, including IL-1β, TNFα and TGFβ (Loots et al., 2012; Weng et al., 2012; Korkosz et al., 2014; Sebastian and Loots, 2017; Al Shareef et al., 2018), can increase Sost and Dkk-1 in bone and cartilage by transcriptional control. This finding could not be reproduced in our study with DPC. Treatment with IL-1β even led to a contrary effect, i.e. shows downregulation of *SOST* and *DKK-1*. This is in line with another publication in fibroblast-like synoviocytes (Yoshida et al., 2018). Thus, pro-inflammatory molecules might have potential to enhance as well as to downregulate Sost and Dkk-1, depending on the cell type. Future studies will have to evaluate, if other oral cell types behave different from DPC in Wnt signaling inhibitor responses under inflammatory conditions. Currently there are hints that periodontitis in gingival tissues increases Sost and Dkk-1 expression (Napimoga et al., 2014).

Tissue mineralization is a characteristic function of bone tissue (Orimo, 2010), but also DPC show mineralization capacity by alkaline phosphatase production and calcium deposition (Müller et al., 2015). Our results confirmed this under normoxia, but under hypoxia mineralization capacity seems to be strongly suppressed. Parallel metabolic activity assays confirmed that an increasing percentage of DPC under hypoxic conditions for 7 and 14 days is metabolically inactive, thus, do not contribute to mineralization.

Gene and protein expression was studied for a shorter period. A treatment duration of 24 h was chosen, because after dental trauma inflammation and hypoxia usually would not be left untreated for longer than 24 h. It is possible that the translation process of SOST and DKK-1 takes more time, which has to be evaluated in further experiments. Even though mRNA levels of *SOST* and *DKK-1* were quantifiable, protein production was not detectable by western blot analysis (Supplementary Figure 1) or enzyme-linked immunosorbent assays (unpublished data), although our previous study was successful in detection of low levels of SOST and DKK-1 (Janjić et al., 2018a). The current study differs in culture conditions from the previous study where cell culture medium contained serum. For this study, only serum-free medium was used to avoid cross-activity with pro-inflammatory molecules. This difference in culture conditions might influence gene expression behavior of the cells. For both studies primary cells from human donors were used, a possibility of patient-specific reactivity has to be considered. In both cases protein expression was hardly detectable or not at all. This suggests that in the dental pulp transcriptional regulation of Sost and Dkk-1 might play a more important role than protein expression, at least under tested conditions. Underlying mechanisms have to be clarified in further experiments.

Taken together, our results show that basal levels of Sost and Dkk-1 in human DPC are very low and often not even detectable. Thus, it would be of interest for experimental purposes to find out if any molecule can stimulate Sost and Dkk-1 production and which function they have in the dental pulp. For clinical purposes, it might be of interest to target Sost and Dkk-1 at a transcriptional level rather than to target protein expression. The hypoxia mimetic agent L-MIM does not seem to have a pronounced effect on *SOST* and *DKK-1* production, while hypoxia significantly downregulates them. Since effects of hypoxia mimetic agents and hypoxia are based on different molecular mechanisms it would be interesting to find out which pathway regulates the decrease of *DKK-1* upon hypoxia treatment. Our previous study gives a hint that hypoxia-inducible factor (HIF) is one component that participates in this mechanism (Janjić et al., 2018a). Our data show that seen effects are independent of the influence of the inflammatory markers IL-1β, TNFα and TGFβ, which do not seem to have a pronounced effect on *SOST* and *DKK-1* in DPC. Finally, if modulations in this study were detected, they were detected at mRNA level only. Therefore, the biological function of the two Wnt signaling inhibitors Sost and Dkk-1 is questionable in the dental pulp, since the molecules would exert their biological activity as proteins.

## Conclusions

Based on our studies on Sost and Dkk-1 in human DPC it can be concluded that hypoxia significantly downregulates *DKK-1* mRNA levels, independent of pro-inflammatory markers. Overall, the protein levels of Sost and Dkk-1 are so low that a biological function of the here observed downregulation is unlikely. Future research will have to assess what exact role Dkk-1 plays in the dental pulp and further how *DKK-1* can be targeted with hypoxia-based approaches for regenerative endodontic strategies.

## Data Availability Statement

The datasets generated for this study are available on request to the corresponding author.

## Ethics Statement

We used an established protocol for cell isolation. When informed and written consent was given extracted third molars were used to prepare fibroblasts from the dental pulp. The protocol was approved by the Ethics committee of the Medical University of Vienna (631/2007).

## Author Contributions

KJ and MS were involved in study design, literature research, data analysis, writing, and submitting the manuscript. AM was involved in the study design, study, and manuscript preparation. HA was involved in study design, data analysis, writing, and submission of the manuscript.

### Conflict of Interest

The authors declare that the research was conducted in the absence of any commercial or financial relationships that could be construed as a potential conflict of interest.

## Supplementary Material

Supplementary Figure 1 Sclerostin (SOST) and Dickkopf-1 (DKK-1) protein levels in human dental pulp cells (DPC), compared to the reference protein glyceraldehyde-3-phosphate dehydrogenase (GAPDH). DPC were treated with interleukin-1beta (**A**; 2), tumor necrosis factor alpha (**B**; 2), and transforming growth factor beta (**C**; 2). Additionally, treatment was combined with L-mimosine (**A–C**; 4) or hypoxia (**A–C**; 6). Untreated DPC (**A–C**; 1), L-mimosine (**A–C**; 3), and hypoxia (**A–C**, 5) were added as control.
