# Supplementary figures and images for: The Influence of Pro-Inflammatory Factors on Sclerostin and Dickkopf-1 Production in Human Dental Pulp Cells Under Hypoxic Conditions
Source: Front Bioeng Biotechnol. 2019 Dec 17;7:430. doi: 10.3389/fbioe.2019.00430 (PMC6927906; doi:10.3389/fbioe.2019.00430)

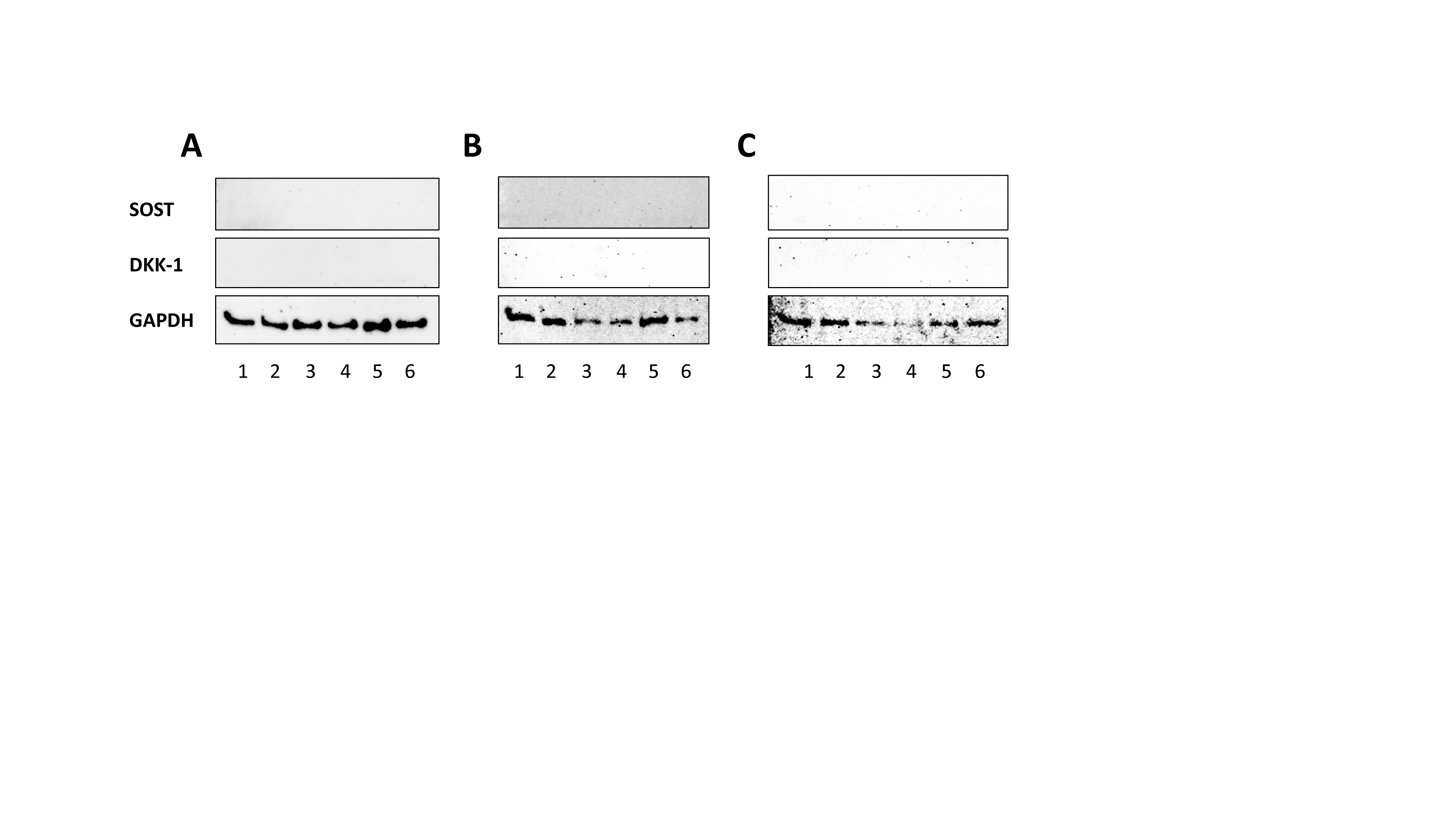

Supplement: Supplementary Figure 1 — Sclerostin (SOST) and Dickkopf-1 (DKK-1) protein levels in human dental pulp cells (DPC), compared to the reference protein glyceraldehyde-3-phosphate dehydrogenase (GAPDH). DPC were treated with interleukin-1beta (A; 2), tumor necrosis factor alpha (B; 2), and transforming growth factor beta (C; 2). Additionally, treatment was combined with L-mimosine (A–C; 4) or hypoxia (A–C; 6). Untreated DPC (A–C; 1), L-mimosine (A–C; 3), and hypoxia (A–C, 5) were added as control. [file Image_1.TIF]
